# A review of children’s participation in disaster risk reduction

**DOI:** 10.4102/jamba.v8i1.218

**Published:** 2016-03-17

**Authors:** Chipo Muzenda-Mudavanhu

**Affiliations:** 1 Department of Geography, Bindura University of Science Education, Zimbabwe

## Abstract

Children are often excluded from disaster risk reduction (DRR) activities, yet they are one of the most vulnerable groups to disasters. As a result, they experience physical, psychological and educational vulnerabilities. There is lack of research on children’s participation in DRR and their potential value in strengthening community resilience has been largely overlooked. Therefore, this article highlights the existing research and knowledge gap in children’s participation in DRR. It highlights the existing research and knowledge gap by reviewing literature on the concept of children’s participation in DRR. The article analyses the different ways in which children’s participation in DRR has been conceptualised, and how this has influenced the way children are involved in DRR. The study will then explore the obstacles to involving children and their potential contribution in DRR.

## Introduction

Children represent the largest segment of the population in developing countries and are often the first victims of natural disasters (Martin 2010). They ‘make up the largest segment of populations affected by disasters (Anderson 2000; Ariyabandu 2000; De Waal, Taffesse & Carruth 2003; Enarson 2000; Fothergill 1996; Gordon, Maida & Farberow 1999; International Federation of Red Cross & Red Crescent Societies 2003; Koger 2006; Morrow & Phillips 1999; UNICEF 2006)’ (Jabry 2005:4). Throughout the world, about 66 million children are affected every year by disasters such as floods, earthquakes and drought, resulting in the violation of their rights (Nikku 2012). Between 1991 and 2000, about 76.5 million children under the age of 15 with 75 million ‘living in developing countries had their lives severely disrupted by natural disasters’ (Babugura 2008:127). Children are often faced with devastating impacts such as limited access to food, shelter, social support, and health care when disasters strike (Babugura 2008). They may be frightened or traumatised, while also being at risk of separation from their families, without forms of identification, and can become potential victims to many forms of exploitation or abuse (Taylor 2014).

In order to better protect and help children, much is still to be learned (Peek 2008):

Although significant progress has been made in understanding children’s mental health needs following disasters, there is still much to be learned about children’s experiences in disasters, their unique vulnerabilities, and their special capacities. (p. 3)

‘While disasters cannot be avoided, the risks faced by children can be prevented or lessened’ (Taylor 2014:77). Given that children are the most affected by disasters and most photographed, it would be expected that their particular vulnerabilities would take priority, which has not been the case (Babugura 2008). Very little research has addressed children’s vulnerabilities and capacities during times of disaster in developing nations (Jabry 2005). ‘Following a disaster, we cannot assume that children’s needs are met if their parents’ needs are met’ without a sustained focus on them (Peek 2008:4). Children are the least listened-to members of society (Jabry 2005) and are ‘rarely given the opportunity to voice their concerns and experiences with disasters’ (Babugura 2008:127). Hence the need to explore the vulnerabilities of children when disasters strike from children themselves (Peek 2008).

Despite recognition of the need for involving children, there is also lack of empirical evidence to support their involvement in disaster risk reduction (DRR). Following the United Nations Convention on the Rights of the Child (UNCRC 1989), four areas of children’s rights were established, namely survival, development, protection and participation. The first three were said to be addressed by legislation, but participation as stated in Article 12 of the UNCRC (1989) is often less supported. Moreover, the way article 12 is translated varies among countries (Lister 2007). Alderson (2001) also notes that many view the participation rights as aspirational and not yet fully realised. Yet children can act as informants within informal community communication networks, convey risk messages with a meaning and have a clear and uncluttered view about risks (SAARC 2011). However, though they can participate in DRR activities, little is known about specific factors that could promote children’s participation in DRR. This review therefore explores the effects of disasters among children, the concept of children’s participation, the obstacles to involving children and their potential contribution in DRR. The following section first examines children’s vulnerabilities and experiences of disasters and the concept of participation.

## Effects of disasters among children

Children are particularly vulnerable to the impacts of natural disasters (Mitchell & Borchard 2014; Seballos et al. 2011). In developing countries, the largest population consists of children who are facing ‘daily risks related to persistent poverty, street crime and violence, poor health, no or low-quality housing, and inadequate and ineffective schools’ (Peek 2008:11), and are continually affected by natural disasters. They are living in crisis before disasters strike (Peek 2008). ‘With disaster frequency and intensity increasing, especially in developing countries (Guha-Sapir, Hargitt and Philippe 2004), combined with poverty and HIV/AIDS, more children will be affected’ (Mason et al. 2005; cited in Babugura 2008:127).

Children’s ‘vulnerability has been explored through the use of statistics that draw attention to their specific vulnerability as an aggregate social group’ (Tanner 2010:340) (Table 1).

**TABLE 1:** Disaster impacts among children.

Year	Place	Disaster	Effect	Reference
2004	India	Indian Ocean tsunami	At least 60 000 children died.	Oxfam International (2005)
2004	Indonesia	Tsunami	1150 schools in Indonesia damaged or destroyed.	UNICEF (2005)
2005	Pakistan	Earthquake	10 000 school buildings collapsed; 16 000 children died in schools that collapsed.	Hewitt (2007); Wachtendorf et al. (2008)
2005	United States’ Gulf Coast	Hurricane Katrina	1100 schools were closed and 372 000 children were left without a school to attend in the chaotic response to Hurricane Katrina, some 2430 children were separated from their families; 370 000 children, were forced to relocate from their homes along the US Gulf Coast; Vital records were lost in the storm, which resulted in delayed enrolment for some youth.	Wachtendorf, Brown and Nickle (2008); Picou and Marshall (2007)
2006	Philippines on Leyte Island	Mudslide	About 200 children were buried alive.	Wachtendorf et al. (2008); ADPC (2007); Peek (2008)
2008	Nepal	Floods	67 schools and 23 000 students.	Dennison and Keim (2009)
2008	Wenchuan in Sichuan, China	Earthquake	12 000 school buildings in Sichuan Province and 6500 school buildings in Gansu Province were damaged or destroyed, disrupting the education of some 2.5 million children.	Peek (2008); UNICEF (2010)
2011	New Zealand, Christchurch	Earthquake	School closures, demolitions, power cuts, and the establishment of temporary school sites.	Mutch (2013); Mitchell and Borchard (2014)

Note: Please see the full reference list of the article, Muzenda-Mudavanhu, C., 2016, ‘A review of children’s participation in disaster risk reduction’, *Jàmbá: Journal of Disaster Risk Studies* 8(1), Art. #218, 6 pages. http://dx.doi.org/10.4102/jamba.v8i1.218, for more information.

Table 1 presented recent catastrophes in which children have been affected. Besides the physical vulnerability, children’s vulnerability also ‘includes psychological factors that can be influenced by loss of family members, material loss, exposure to disaster, low levels of social support, and displacement’ (Mudavanhu et al. 2015:268). Children’s homes, neighbourhoods and schools have been destroyed and, in most cases, lives have been lost but they represent an understudied group and their challenges are often overlooked.

According to Mudavanhu (2014):

The loss of livelihood from disasters can lead to extreme poverty and may lead to school dropouts and malnutrition. Dropping out of school may lead to early marriages for the affected children, especially girls, and will result in them being trapped in a vicious cycle of poverty. (p. 1)

Recurring disasters do not only affect a child’s basic right to live (Article 6 of UNCRC), health (Article 24); and education (Article 27), but they cut across their right to participate (Article 12) and for decisions to be made in their best interests (Seballos et al. 2011). Exposure to disasters can be a traumatic experience for children, affecting future full development potential (ADPC 2007) and the way disasters affect the lives of children creates an urgent need for evidence-based research to explore the experiences of children in already marginalised communities, and how the conceptualisation of children’s participation plays in their potential contribution to DRR. Addressing these will allow children’s vulnerabilities to be correctly addressed by policy makers to reduce the disaster impacts and strengthen community resilience. Therefore, the next section explores how children’s participation has been conceptualised and the potential role in DRR.

## Conceptualising children’s participation

Despite readily available information and knowledge about how children are caught up as victims of disasters, little work focuses on children as agents of change in the prevention or mitigation of disasters in developing countries. Children are viewed as passive victims during emergencies. An alternative approach is to go beyond the immediate impact of disaster, the emergency phase and look into the role of children to withstand, manage as well as cope with disaster risk and reduce vulnerability through capacity-building, education and awareness. This goes beyond the extent of current research that tends to ‘assume that children are passive victims with no role to play in communicating risks, participating in decision making processes’ and ‘preventing disasters’ (Mitchell et al. 2008:255).

Participation is said to be held up as a panacea for all the problems faced by children in developing countries (Skelton 2007). Whilst children’s participation is recognised in the development process, its recognition in DRR is an emerging concern and is achieving increased attention as a component of DRR (Lopez et al. 2012). The idea of children’s participation is in line with international commitments towards child rights (Mitchell et al. 2008). International declarations, treaties and conventions were designed to cater for the needs of children (Table 2).

**TABLE 2:** Treaties and declarations for children.

Year	Conference	Purpose
1924	Geneva Convention	Recognises that mankind owes to the Child the best that it has to give, declare and accept it as their duty that, beyond and above all considerations of race, nationality or creed.
1959	UN Declaration of the Rights of the Child	A child, by reason of his physical and mental immaturity, needs special safeguards and care, including appropriate legal protection, before as well as after birth.
1948	UN Universal Declaration of Human Rights	Provides for participation rights for all people, including children.
1966	UN The International Covenant on Civil and Political Rights	Provides for participation rights in articles 18, 19, 21, 22 and 25(1), which are clear on and fundamental to the fact that children have the right to participate in matters affecting them and their well-being.
1989	UN Convention on the Rights of the Child	A universally agreed set of standard and obligations to protect the human rights of children.
1992	United Nations Conference on Environment and Development	Produced Agenda 21 and a Programme of Action which states that children are a major group who should be part of participatory processes for sustainable development and environmental improvement.
1996	The Habitat Agenda from the United Nations Conference on Human Settlements	States that government must utilise participatory approaches which include intergenerational interests relating to sustainable human settlements demanding special attention to children.
2000	The United Nations Millennium Declaration	Undertakes to ensure a high level of participation for all citizens, including children, in order to achieve the Millennium Development Goals.

The United Nations (UN) conferences were trying to address children’s complex issues. Though there was some participation in some of the conventions, the concept of children’s participation received attention in the 1990s following the UNCRC (1989), which stated that children and young people should have their opinions taken into account in all major decisions affecting their lives (Article 12) (Skivenes & Strandbu 2006). Before then, the emphasis was on provision and protection and not on participation rights. The UNCRC is the most ratified international convention (Mitchell et al. 2008) and it marked a significant shift in thinking about children and has introduced participation as a third ‘P’ alongside with ‘Provision’ and ‘Protection’ (Skelton 2007). He further noted that the UNCRC marked a change in approach to the children’s world.

Mitchell, Tanner and Haynes (2009) defined children’s participation as involving children in responsible, challenging action that meets their needs, with opportunities for planning and decision-making affecting others in an activity whose impact or consequence is extended to others. Save the Children (2000) defined children’s participation as children sharing ideas, thinking for themselves, expressing their views effectively, planning, prioritising and being involved in the decision-making process. Children’s participation is also a process in which children have real influence in the decisions that affect their lives and not just a token or passive presence in adult agencies (Mitchell et al. 2008). By participating in community activities, children can define what they perceive to be problems, rather than having to accept issues that have been identified and mediated by adults or authorities (Mitchell et al. 2008). Martin (2010) views children’s participation as a right in itself and a means to ensuring children’s protection, survival and development.

Viewing participation as an end in itself creates a meaningful experience for children and Reddy and Ratna (2002) described it as transaction participation. This type of participation creates ownership, opportunities and possibilities for children. Creating real opportunities for children to experience meaningful participation regardless of the objective of the activity is a goal in itself (Naker 2007). But, is it a question of creating an opportunity, ownership and possibility for children? What exactly do children require for their participation to be effective and their voices to be heard in times of crisis? Following the outcome oriented might have a danger of having children’s participation as tokenism which is regarded as non-participation.

A closer look at the definitions also suggests that children’s participation is a means to an end and not an end in itself. Most definitions view children’s participation as process oriented and not outcome oriented. Kirby et al. (2003) noted that meaningful participation must be seen as a process not simply an isolated activity or event. (Naker 2007) also supports this by defining it as not an end but a means to an end where empowerment is the outcome. He went on to say that participation should be seen as a continuum, emphasising it as a process, rather than just the outcome. Thus, children’s participation should not be about selecting or inviting a few children to represent others but contributes to realising development, survival and protection (Skivenes & Strandbu 2006). Development, survival and protection enable children to participate and realise the right to life, health and education. Viewing children’s participation as a process or means to an end emphasises the role of children in disasters. This approach views empowerment as an outcome of participation. Empowerment in this case means providing opportunities and experience, to allow children to be actively involved in decision-making about issues that affect them at different levels (UNICEF 2011). This enhances feelings of control, meaning and connectedness contributing to community resilience (Oliver et al. 2006).

Although children’s participation gained momentum in the past decade and is now in everyday use, its actual meaning is complex. The concept of participation is contested, broad and not clearly defined (Barbar 2009; Fleming 2012; Gallagher 2008).

According to Skelton (2007):

Recent perspectives on children’s participation have emerged from a range of fields: global discourses and conventions; individual countries and state legislative practices; development discourses and actors; academic paradigm shifts; and children themselves. (p. 169)

Thus, how participation is defined depends on whether it is regarded as a principle, practice or an end in itself. Now the question is whether children’s participation is a process or an end in itself. The ‘major challenge is identifying what children’s participation exactly’ mean ‘and the requirements for it to be fulfilled’ (Skivenes & Strandbu 2006:11). Answering this question helps us to understand how children’s participation can be meaningful and its potential role in DRR.

## Children’s potential role in disaster risk reduction

‘Reviews of children’s participation in DRR have shown that they’ yielded ‘positive outcomes’ for children and their communities (Lopez et al. 2012). In Mozambique, children have developed a ‘greater knowledge of risks and how to’ minimise ‘it through their participation in DRR activities’ (Back, Cameron & Tanner 2009). Another study in South Asia ‘showed that through incorporation of children’s participation in disaster preparedness, rescue, rehabilitation and relief phases a community’s ownership and sustainability can be enhanced’ (Nikku 2006 cited in Lopez et al. 2012:304). Children who participated in DRR activities, according to Mitchell et al. (2008), increased their level of confidence in responding to disaster risk.

Children are innovative agents of change only if they are involved. They can maximise the adaptive capacity needed to address disaster risk. ‘When equipped with relevant knowledge and skills’, children can make informed decisions. For example, in the:

Philippines, children from the Camotes Islands have worked together to restore degraded mangrove ecosystems by assembling teams to collect and replant tree saplings in sanctuaries with the establishment of protective barriers. They combined local knowledge with a range of expert resources to raise awareness of the multiple benefits of mangrove restoration. The benefits include livelihood gains through provision of aquatic spawning grounds, maintaining biodiversity, protection from typhoons, rising sea levels, wind and surge risks. Young people drew in community members and succeeded in mobilising constituencies to ensure the greater protection of their ecosystems. (Plan 2010:10)

Furthermore, in the Philippines, children ‘worked together with adults to restore degraded mangrove ecosystems resulting in livelihood gains’, providing ‘spawning grounds, biodiversity gains, disaster protection from typhoon winds and storm surges, adaptation to climate change impacts’, and the removal of atmospheric greenhouse gases causing climate change (Tanner et al. 2009:58).

According to Plan (2010):

They shared their views at community meetings, used local media to raise awareness and distributed simple yet scientifically sound information, education and communication materials stressing the importance of offsetting the impacts of climate change by protecting the community’s natural resources (p. 35)

In developing countries, children take the responsibility of doing household chores and contributing to agricultural work (Liebel & Saadi 2010), but it is difficult for many organisations and societies to fully accept that children can take responsibilities in DRR. Lansdown (2010) also notes that children’s rights to negotiate their contributions are highly restricted. As a result, many projects do not succeed because children are treated as bystanders rather than active participants (Roshani 1997). Therefore, for participation to be effective, it must become embedded in institutions and processes that influence children’s everyday lives (O’Donoghue, Kirshner & Mclaughlin 2003).

Although children’s ‘participation is not a replacement for adult responsibility, empowering children through their participation is an important protection strategy’, as well as a right (Save the Children 2006:2). Children’s participation in DRR would ensure their safety. This is supported by Plan (2010), who also added that participation and involvement in DRR fosters the agency of children to work towards making their lives safer and their communities more resilient. Because children are the most vulnerable group, during disasters, they need to be encouraged and motivated to participate in making the world a safer place to live. According to Izadkhah and Hosseini (2005:142), ‘the theme of the United Nations 2000 World Disaster Reduction Campaign was “Disaster Reduction, Education and Youth”’ aiming ‘at the continuation and development of a culture of prevention through education so that children can take a pro-active role in understanding risks and reducing the impact of disasters.’ The campaign (2001) stated that:

A culture of prevention is something that forms over time. What is needed is a change of attitude, based on the conviction that we do not need to be fatalistic about disaster risks and a willingness to act upon that conviction. The mind-set is best developed at any early age. (Izadkhah & Hosseini 2005:142)

Children’s participation in DRR empowers them to make informed decisions concerning the risks of disasters. Though investing in child-focused DRR is a long-term process, it creates a generation that is better prepared for the disasters of tomorrow. Apart from empowerment of children through DRR, their involvement contributes to the realisation of their rights. This was also supported by Mitchell et al. (2008), who pointed out that the approach also recognises children as key actors in their own development and in their communities. Children’s participation in DRR is also an entry point for programmes aiming at promoting sustainable development and children’s rights.

Some immediate examples that can be utilised to enhance the participation of children in disasters is that children with illiterate parents can convey messages about DRR. They can also recognise disasters alongside social and economic threats (Mitchell et al. 2008). Though they can convey disaster messages and recognise disasters, they are usually not given the chance to do so. Given the resources, encouragement and the opportunity to participate, there is also a need to determine the manner in which children can build community resilience in their areas. What exactly are children expected to do in the society to build community resilience and at the same time reduce their own risks?

‘While there is a growing evidence that taking a child-centred approach to community based adaptation can build the adaptive capacity of children and also provide benefits to entire communities, there is no evidence’ to prove ‘that what has worked in a growing number of cases is more broadly applicable, translatable to other regions or sustainable in the absence of direct project support’ (Mitchell & Borchard 2014:372).

## Risks and limitations of involving children in disaster risk reduction

Though there is a long history behind children’s rights in international agreements, there is a gap between the rhetoric of the agreements and reality of authorities’ provisions for children (Chawla 2002). Countries are finding it difficult to make children participate because (1) they lack clarity as to what participation means (2) there is lack of legislation (3) adult and cultural resistance (4) lack of capacities and (5) lack of monitoring and evaluation tools.

Children are different from adults. Engaging children in DRR may be thwarted by uneven motivation to participate. ‘Since these children are under’ the custodian of ‘the adults the family and community context in which they live can present barriers for their engagement’. Children are children, ‘some may show interest whereas others are unsure and may lack support from adults to proceed (Shaw 2006). Children might find it difficult to work on their own’, they ‘need supporting environment’, which is difficult to provide because politics does not consider the views of children because they are non-voters. In addition, Peek (2008) also noted that children’s knowledge of risk and disasters differs across cultures, physical and social environments, and family structures. As a result, not all children have the same strengths or abilities. Though children can be involved only in age- and culture-appropriate activities, the results may not be the same.

This requires experts’ commitment and political will (Peek 2008):

This means that activities must be evaluated beforehand to ensure that they are suitable for the ages and capacities of the children involved and so that risky or potentially hazardous activities are avoided. (Raftree *et al*. 2002; cited in Peek 2008:18)

There may also be a lack of commitment by decision-makers to accept children’s views and a failure to represent them. In most developing countries, communities do not believe in children’s rights but rather that children should follow what the elders say. This poses some challenges to fully involving children in DRR activities. Parents sometimes hinder their children from participating; some fear losing control over their children while others do not trust their children’s capabilities. Some of the factors that also affect children’s participation in DRR include: the availability of resources to mobilise participation, staff training and development and clarity and shared understanding about the objectives and outcomes of their participation.

Disaster risk is also a complex issue involving the physical environment, and the social, cultural, political and economic spheres of the society. This complexity is the major obstacle to effective children’s participation in DRR. A holistic approach can be applied for effective DRR, but that option has failed to influence policy makers in most developing countries.

## Conclusion

It is noteworthy that the concept of children’s participation in DRR does not imply wiping away childhood, treating children as adults or pressurising them to make choices. Rather children’s participation may be a way of encouraging them to be involved in issues that concern their lives in order to reduce their vulnerability. The value of children’s knowledge, creativity, energy, enthusiasm, and social networks can also be recognised and encouraged through their participation. It then follows that, if children’s participation is about encouragement, is it possible to encourage participation and reduce disaster risk using non-structural measures in communities, so as to avoid the aid dependency syndrome? The aid dependency syndrome has been flagged as an anti-developmental process.

It would also be useful to know how children’s participation in DRR affects the future. If it affects the future, how possible is it to incorporate DRR programmes in disaster risk education as part of the school curricular, co-curricular or community activities that foster awareness and better understanding of the immediate environment in which children and their families live and work? There is therefore a need to understand the knowledge and education nexus in order to build a culture of safety and resilience at all levels of the community.
